# Regional variation in access to and quality of acute stroke care: results of Germany’s Health System Performance Assessment pilot, 2014–2020

**DOI:** 10.1007/s43999-024-00045-x

**Published:** 2024-07-02

**Authors:** P. Hengel, U. Nimptsch, M. Blümel, K. Achstetter, R. Busse

**Affiliations:** Department of Health Care Management, Technische Universität Berlin, Str. des 17. Juni 135, Berlin, 10623 Germany

**Keywords:** Health System Performance Assessment, Regional variation, Unwarranted variation, Stroke unit, Quality of care, Germany

## Abstract

**Background:**

Health System Performance Assessments (HSPA) and analyses of unwarranted regional variation in health care both aim at identifying strengths and weaknesses of health systems to improve care. Applying HSPA’s conceptual approach of interrelated health system dimensions (e.g., access, quality) to regional levels might help to better understand variation in care to reduce inequity and improve performance.

**Methods:**

We use four indicators identified and analysed in a pilot study for a German HSPA to assess variation in access to and quality of acute stroke care between Germany’s 16 federal states and urban vs. rural regions from 2014 to 2020. Stroke unit (SU) density, share of the population reaching a SU within 30 min by car, share of inpatient stroke cases treated in a hospital with a SU, and inpatient mortality were computed based on hospital quality reports and discharge data covering all acute care hospitals. Inpatient mortality was adjusted for age, sex, stroke type, and comorbidities.

**Results:**

About 500 SU were identified, i.e., 2.0 per 1,000 inpatient stroke cases. Almost 95% of Germans could reach a SU hospital within 30 min (rural: 90%; urban: 99%; > 88% in all states but one). The share of inpatient stroke cases treated in a SU hospital increased to 93% with a decreasing span between rural (92%) and urban (95%) regions and between states (74–98%). Inpatient mortality stagnated around 8.5% and increased to 9.0% in 2020 (rural: 8.7%; urban: 9.2%; states: 7.0–9.7%, one outlier of 13.3%).

**Conclusions:**

The results especially revealed varying performance patterns in access to and quality of acute stroke care between the federal states, indicating different areas for improvement which might be addressed by more targeted policy measures in the future.

**Supplementary Information:**

The online version contains supplementary material available at 10.1007/s43999-024-00045-x.

## Background

Strengthening health systems is a major lever towards the ultimate goal of reaching a better health for all within a society. In this regard, Health System Performance Assessments (HSPA) can provide evidence for a deeper understanding of how and how well health systems work. In HSPA, health systems are monitored and evaluated in a systematic and continuous manner based on their achievements in overarching goals, or health system dimensions (e.g., access to care, quality, population health, health system responsiveness, or efficiency) [1, 2]. Reviewing outcomes over time and between as well as within countries thereby provides a picture of the development and performance of a health system. A specific strength of HSPA is its broad perspective on health care, combining health system dimensions and allowing to analyse their interrelations with regard to overall performance. While in general, the systemic perspective on health system activities in HSPA also includes coverage of all areas of service provision, diseases, and population groups, HSPA outcomes can also be used to zoom in on specific strands of care, e.g., a certain field of care provision like stroke care. This is especially of relevance for exploring underlying reasons for macro level health system performance to identify areas for further improvements, and to ideally generate actionable evidence for policy.

Similarly, HSPA results might also be viewed on a regional level within a country, both to identify possible intra-societal inequities and because health care is often at least partly organised regionally and therefore this is where information to possibly act upon is needed. In Germany, for instance, health care organisation is split between the national level, the level of the federal states, and the municipalities, each of which have different competencies [3]. This aspect of HSPA is closely related to assessments of regional variation in health care as done in, e.g., health care atlases [4]. Such analyses seek to identify unwarranted variation between regions, i.e., “variation that cannot be explained by the condition[s] or the preference[s] of the patient[s]; it is variation that can only be explained by differences in health system performance” ([5] as cited by [6]). While health care atlases often focus on or exclusively consider utilisation of services, broadening this perspective to other dimensions of health systems might help to better understand regional variation and to identify the areas most important for improving health care and health systems as a whole [4].

In a pilot study for a German HSPA, a conceptual framework was developed based on a review of existing frameworks (e.g., [1, 2, 7]) consisting of ten health system dimensions, including access to and quality of care [8]. Access to the health system and to care was defined as the possibility and ability to obtain care when needed and was divided into three steps, or sub dimensions, based on existing literature (e.g., [9–11]): availability of services and providers, geographical accessibility, and affordability of services. Quality of health care was conceptualised as the efficacy of the obtained care, i.e., the extent to which the overall aim of health care – the preservation or improvement of health – has been achieved [12, 13]. Quality indicators were distinguished as structure, process, or outcome quality [14, 15]. To measure performance, indicators were identified for each dimension based on the international literature, and secondary data sources were used for analyses to facilitate a regular assessment. Next to computations for Germany as a whole, indicators were also calculated for subgroups within Germany. These equity analyses included regional levels like federal states and urban vs. rural regions, next to socio-economic groups and others. While indicators were generally chosen to reflect the respective health system dimension covering all sectors and diseases, specific (groups of) diseases were addressed in sub indicators. Among those are four indicators on access to and quality of acute stroke care.

The aim of this article is to assess regional variation in access to and quality of acute stroke care in Germany based on the results of Germany’s HSPA pilot study. Stroke is among the diseases contributing the most to the burden of disease in Germany, being responsible for 4% of all disability-adjusted life years (about 6% of years of life lost and 2% of years lived with disability) [16, 17]. In acute stroke care, prompt access to an adequately equipped site, mostly a stroke unit (SU), is vital [18–20]. As the federal states are responsible for hospital planning in Germany, differences in access to and quality of acute stroke care on a regional level might indicate inequalities in care provision attributable to governance. Therefore, the analyses specifically seek to answer the following research questions:

1. How big is the unwarranted regional variation in access to and quality of acute stroke care in Germany at the level of the 16 federal states and in rural vs. urban regions?
2. How did the regional variation develop over time, from 2014 to 2020?
3. How do the results differ on the level of the federal states within and between indicators?
4. Which – if any – implications for policy can be drawn from the results?

## Methods

The following four indicators on access to and quality of acute stroke care were identified and analysed in the pilot study for a German HSPA: (1) SU density, to capture availability of acute stroke care as part of the access dimension; (2) share of the population reaching a SU within 30 min by car as geographical accessibility of acute stroke care; (3) share of inpatient stroke cases treated in a hospital with a SU as an indicator of process quality; and (4) inpatient mortality as an indicator of outcome quality.

### Data sources

Two data sources were used for indicator calculations: hospital quality reports and German Diagnosis Related Groups (DRG) statistics. Hospital quality reports are mandatory for all acute care hospitals and are publicly available. They cover general data such as hospital location, staffing, number of beds, and case volume by both principal diagnosis and procedures [21]. DRG statistics are nationwide discharge data covering all inpatient cases in acute care hospitals except for psychiatric and military hospitals. They can be requested from the Research Data Centre of the Federal Statistical Office via remote access [22, 23]. DRG statistics include case data on diagnoses, procedures, and demographics, among others, but no patient identifier to assign (re)admissions to a single person. In contrast to hospital quality reports, DRG statistics also do not include hospital addresses necessary to calculate SU density, which is why the two separate data sources were used. Additionally, hospital quality reports refer to hospital sites separately, while in DRG statistics, hospital identifiers may include more than one site.

### Operationalization of indicators

For all analyses, inpatient stroke cases were defined as cases aged 20 years and over with a principal diagnosis of either subarachnoid haemorrhage (I60), intracerebral haemorrhage (I61), cerebral infarction (I63) or stroke, not specified as haemorrhage or infarction (I64) according to the International Statistical Classification of Diseases 10 German Modification (ICD-10-GM). Cases transferred from other hospitals (identified via admission code) were excluded from the analyses for comparability between regions (see below).

SU density was defined as number of SU per 1,000 inpatient stroke cases. SU were identified in hospital quality reports as hospitals with at least ten SU procedure codes per year (German Classification of Operations and Procedures, Codes 8-981 and 8-98b), as done in previous studies [20, 24]. Number of inpatient stroke cases was derived from DRG statistics and was used as denominator to account for needs-adjustment between regions.

To obtain the share of the population reaching a SU within 30 min by car, travel time to the nearest SU was computed and provided by the Federal Institute for Research on Building, Urban Affairs and Spatial Development using the national German accessibility model and address data of the hospitals with a SU previously identified in the hospital quality reports [25, 26]. The starting points for the route calculations are over 120,000 measuring points in the traffic network, which are based on the populated 1x1km grid cells. The calculations were carried out with the Network Analyst from ESRI-ArcGIS.

The share of inpatient stroke cases treated in a hospital with a SU on all inpatient stroke cases was calculated using DRG data. As before, hospitals with at least ten SU procedure codes per year were defined as hospitals with a SU. By excluding transfers from other hospitals, only cases with direct admission to a SU hospital and thus having the chance for a timely SU treatment were included to evade bias. Since it depends on many additional factors whether a patient actually receives SU care in a respective hospital, the admission to a hospital with a SU in general was used as an indicator of unwarranted variation in process quality.^1^ Therefore, the indicator assesses the adequate allocation of patients.

Inpatient stroke mortality was defined as the share of stroke cases with a discharge code for death on all inpatient stroke cases in DRG data. Next to crude rates, adjusted rates were calculated using generalized logit regression models for each year separately while treating hospitals as clusters. Age (in years), sex (female/male), type of stroke (I60/I61/I63/I64), and 14 separate secondary diagnosis groups for comorbidities (yes/no, respectively) were included as control variables [20, 27, 28].^2^ Standardized mortality rates (SMRs) were computed as observed number of deaths divided by expected number of deaths (i.e., cumulated probabilities as derived from regression models). Next, adjusted mortality rates were calculated by multiplying SMRs with the crude mortality rate for Germany as a whole, respectively. Here, excluding cases transferred from other hospitals was necessary to account for differences in provision of rehabilitation care between federal states, as it is provided either in acute care hospitals or in specialized hospitals, and both transferral patterns and mortality vary between both. Regression models were fitted for each year separately to draw conclusions about trends in regional variation. That is, reported mortality results are adjusted for differences between regions but not over time. Additionally, one model was fitted for all years, including all mentioned covariates plus calendar years as a categorical variable to allow inferences about mortality trends over time.

### Statistical analyses/presentation of results

Indicators were analysed for Germany as a whole, its 16 federal states, and urban vs. rural regions, respectively. Years 2014 to 2020 are covered for all indicators except for accessibility of SU by car, which was computed in 2023 with SU data of 2020 only. Degree of urbanization was operationalised based on a classification of the Federal Institute for Research on Building, Urban Affairs and Spatial Development, dividing Germany in 96 either urban regions, regions with an increasing degree of urbanization or rural regions, based on population density and sizes of cities [29]. For the analyses, the latter two categories were summarized as being rural regions, in contrast to the first category of urban regions. In DRG data, the assignment of inpatient cases to the respective regions was based on the patients’ places of residence for SU density, while it was based on hospital locations for treatments in SU hospitals and mortality, respectively. Using hospital sites instead of patient residence for the two quality indicators was done to make the results more relevant for policy, because the federal states are responsible for hospital planning within their borders.

Results are presented by region and calendar year, both as absolute values and as relative deviation from the value for Germany as a whole in % (i.e., for mortality, these are SMR values minus 1). Ranges of deviation are used to quantify regional variation. In addition to trends over time and variation between the federal states overall, indicator results are also depicted in maps of Germany to better compare the states to each other both within and between indicators. Further results on the indicators covering all years and all regions are depicted in the Appendix. To assess the variation of results between federal states over time, Kendall’s coefficient of concordance (Kendall’s *W*) was calculated per indicator, i.e., the correlation of the states’ ranks between the years. Alpha was set at .05. DRG data were analysed using SAS Version 9.3 via remote access [30], and further data processing, including preparation of figures, was done in R [31, 32].

## Results

### Stroke unit density

**Fig. 1:**
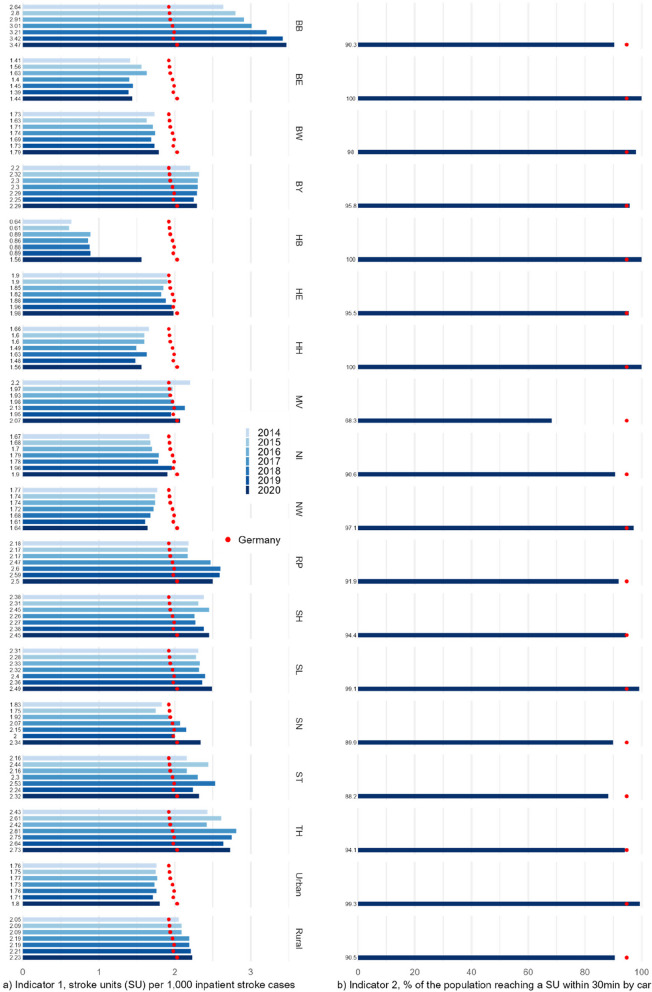

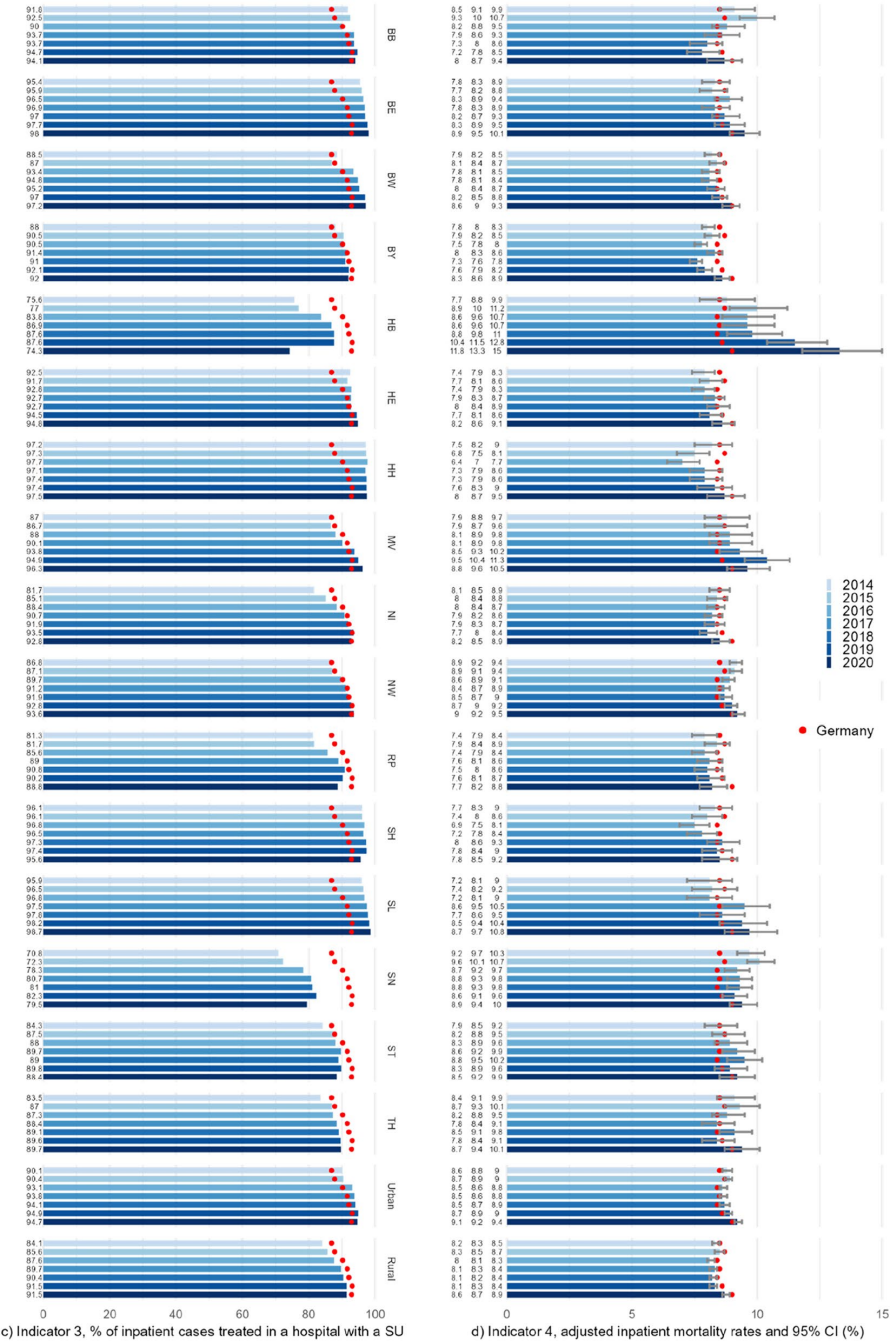
**a**-**d** Absolute values of the four access and quality indicators: frequencies for Germany as a whole, the 16 federal states, and urban and rural regions, 2014–2020. Values on adjusted mortality are derived from regression analyses for each year separately, adjusted for age, sex, stroke type, and comorbidities, i.e., accounting for regional variation but not for time variation; SU – stroke unit, CI – Confidence Intervals, GER – Germany as a whole, BB – Brandenburg, BE – Berlin, BW – Baden-Württemberg, BY – Bavaria, HB – Bremen, HE – Hesse, HH – Hamburg, MV – Mecklenburg–Western Pomerania, NI – Lower Saxony, NW – North Rhine-Westphalia, RP – Rhineland-Palatinate, SH – Schleswig-Holstein, SL – Saarland, SN – Saxony, ST – Saxony-Anhalt, TH – Thuringia

The number of hospitals providing SU care in Germany rose from 477 in 2014 to 506 in 2017, decreasing to 500 in 2019 and 485 in 2020 (Table 1). SU density per 1,000 inpatient cases slightly increased from 1.92 to 2.03 for Germany as a whole between 2014 and 2020 (Fig. 1a). In the federal states, SU density ranges from 0.6 to 3.5 for all years, with (partially strong) increases in about half of the states over time, while a significant decrease is seen in no state. SU density is higher in rural regions throughout all years and increased between 2014 and 2020, in contrast to urban regions.

**Table 1:** Basic characteristics and indicator results of stroke care, Germany as a whole and minimum/maximum values at the federal state level, 2014–2020

	**2014**	**2015**	**2016**	**2017**	**2018**	**2019**	**2020**
**Inpatient stroke cases**							
Germany	248,458	253,501	257,433	256,594	252,902	252,843	238,384
Min.–Max.	3,121–53,606	3,297–54,747	3,352–55,836	3,500–55,272	3,412–54,877	3,353–54,626	1,921–51,854
**Hospitals providing SU care**							
Germany	477	489	500	506	503	500	485
Min.–Max.	2–95	2–95	3–97	3–95	3–92	3–88	3–85
**SU density per 1,000 inpatient stroke cases (Indicator 1)**							
Germany	1.92	1.93	1.94	1.97	1.99	1.98	2.03
Min.–Max.	0.64–2.64	0.61–2.80	0.89–2.91	0.86–3.01	0.88–3.21	0.89–3.42	1.56–3.47
**Share of the population reaching a SU within 30 min by car in % (Indicator 2)**							
Germany	n.a.	n.a.	n.a.	n.a.	n.a.	n.a.	94.7
Min.–Max.							68.3–100.0
**Share of inpatient stroke cases treated in a hospital with a SU in % (Indicator 3)**							
Germany	86.89	87.80	90.19	91.59	92.11	93.12	92.94
Min.–Max.	70.79–97.23	72.29–97.32	78.32–97.71	80.73–97.54	81.02–97.82	82.28–98.16	74.28–98.73
**Inpatient mortality rate in % (Indicator 4)**^**a**^							
Germany	8.55	8.67	8.37	8.45	8.45	8.56	8.97
Min.–Max.	7.87–9.75	7.46–10.13	7.03–9.59	7.78–9.58	7.57–9.85	7.92–11.52	8.22–13.33

*SU* stroke unit, *n.a.* not available

^a^Values on adjusted mortality are derived from regression analyses for each year separately, adjusted for age, sex, stroke type, and comorbidities, i.e., accounting for regional variation but not for time variation

**Fig. 2:**
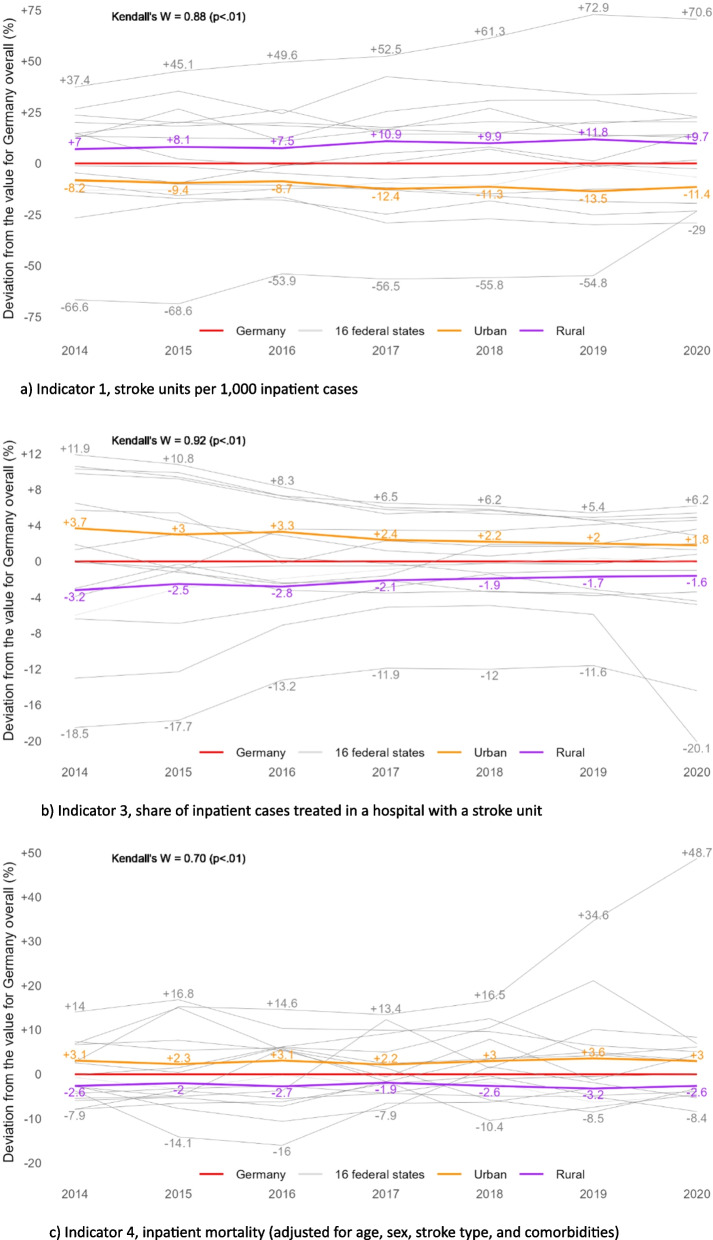
**a**-**c** Relative deviation from values for Germany as a whole in the 16 federal states and in urban vs. rural regions, 2014–2020, for indicators 1, 3, and 4 (no trends available for indicator 2) (Values for all indicators, regions, and years can be found in the Appendix)

In terms of relative deviation from the values for Germany as a whole, the spread between urban and rural regions widened from -8.2% vs. +7.0% in 2014 to -11.4% vs. +9.7% in 2020, respectively (Fig. 2a). For the federal states, the minimum and maximum values shifted from -67%/+37% in 2014 to -29%/+71% in 2020. That is, compared to the value for Germany as a whole, SU density was 29% lower in the federal state with the lowest, and 71% higher in the state with the highest density in 2020 (see also Appendix, tables A1). Kendall’s *W* is 0.88 (*p* < .01), indicating little change in the states’ ranks over time.

### Share of the population reaching a stroke unit within 30 min by car

For indicator 2, values are only available for 2020. In this year, 94.7% of the German population could reach a SU within 30 min by car (Fig. 1b). In three of the 16 federal states, this was true for 100% of the population. The lowest rate was 68.3%, while all other states ranged above 88%. Regarding the degree of urbanization, the share was lower in rural compared to urban regions (90.5% vs. 99.3%).

**Fig. 3:**
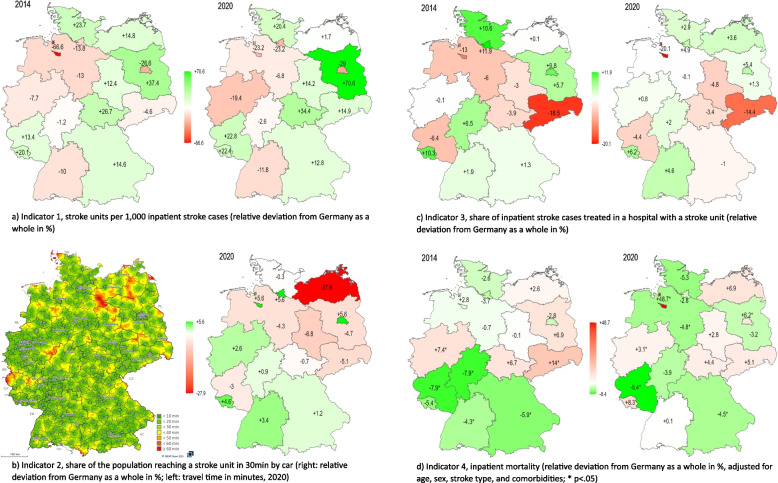
**a**-**d** Maps of Germany for all four indicators showing the relative deviation of the federal states from the values for Germany as a whole (%), 2014 and 2020 (except for **b**)

In relative terms, the federal states range between -6.8% and +5.6% with the one outlier of -27.9%, while deviation from Germany as a whole is -4.4% in rural and +4.9% in urban regions (Fig. 3b and Appendix, tables A2).

### Share of inpatient stroke cases treated in a hospital with a stroke unit

The number of inpatient stroke cases in Germany rose from about 248,500 in 2014 to 257,400 in 2016, followed by a decline to 252,800 in 2019 and 238,400 in 2020 (Table 1). The share of inpatient stroke cases treated in a hospital providing SU care increased steadily from 86.9% in 2014 to 93.9% in 2019 and stagnated in 2020 (92.9%; Fig. 1c). In the federal states, lowest values were below 75% in two states and up to 98% in others. A general positive trend between 2014 and 2019 is seen in all states, which continued in some in 2020 but reversed in others. Rates are higher in urban regions for all years (90.1–94.7%), compared to rural regions (84.1–91.5%), but the latter showed a stronger increase.

Thus, the range between urban and rural regions regarding their relative deviation from Germany as a whole narrowed down from -3.2%/+3.7% in 2014 to -1.6%/+1.8% in 2020 (Fig. 2b). In the federal states, the span was -18.5%/+11.9% in 2014 and reached its minimum in 2019 at -11.6%/+5.4%, widening again in 2020 (-20.1%/+6.2%; see also Appendix, tables A3). Kendall’s *W* is .92 (*p* < .01).

### Inpatient mortality

Of the roughly 250,000 inpatient stroke cases per year in Germany, about 21,000 to 22,000 deceased during their stay, corresponding to a stagnating crude mortality rate between 8.4% and 8.7% from 2014 to 2019, increasing to 9.0% in 2020 for Germany as a whole. In the regression model for all years combined including the calendar year as a categorical variable, only 2020 shows an odds ratio (OR: 1.10) that differs statistically significant from 2014, indicating mortality was elevated in 2020 compared to prior years even when accounting for differences in patient characteristics. In the federal states, adjusted rates range from below 8% in some states up to above 10% in others, with a maximum of 13.3%, reached in 2020. While some states continuously show mortality rates below/above average in all years, no state does so on a statistically significant level (Fig. 1d; regression models for each year separately). In urban regions, adjusted mortality is about 0.2–0.3%-points higher compared to Germany as a whole in all years on a statistically significant level and vice versa in rural regions. Further results of all regression models, including crude and adjusted mortality rates, SMR, and OR of control variables, can be found in the Appendix, tables A4.

In terms of relative deviation from values of Germany as a whole, regional variation increased from -7.9%/+14.0% in 2014 to -16.0%/+14.6% in 2016, and to -8.4%/+48.7% in 2020 (Fig. 2c). The strong increase in 2020 is especially influenced by one outlier, while all other states show no increased variability. Variation between urban and rural regions remained largely stable with a minimum of -1.9%/+2.2% in 2017 and a maximum of -3.2%/+3.6% in 2019. Kendall’s *W* is .70 (*p* < .01).

### Indicator results on the state level

In terms of variation within each indicator over time, the maps in Fig. 3 illustrate the similarities of the states’ results relative to Germany as a whole between 2014 and 2020, as already shown by values of Kendall’s *W*. The biggest variation between states is seen for SU density (about ±70% across all years, Fig. 3a). Here, densely populated states such as the three city-states show the lowest values. The opposite is the case for indicator 2, share of the population reaching a SU within 30 min by car (Fig. 3b, right map). Variation within this indicator is comparatively small, except for one state (-27.9%, rest about ±6%). When looking at the travel times in minutes (Fig.3b, left map), there were some regions in Germany where people needed more than 1 h to the next SU when using a car. Those regions seem to be especially near borders, both between states and to neighbouring countries. For indicator 3, share of inpatient stroke cases treated in a hospital with a SU (Fig. 3c), some similarities are seen in the pattern between the states, compared to indicator 2. In some states, however, the results are of opposite direction. Overall, the states range between +12% and -20% for indicator 3, compared to -8% and +14% (and one outlier of +49%) for indicator 4, inpatient mortality (Fig. 3d). As before, the patterns of state results seem similar between both indicators, but exceptions apply.

## Discussion

This study assessed the unwarranted regional variation in access to and quality of acute stroke care in Germany using 2014 to 2020 data on four indicators of the German HSPA pilot: SU density per 1,000 inpatient stroke cases to capture availability of providers; the share of the population reaching a SU within 30 min by car for geographical accessibility; the share of inpatient stroke cases treated in a SU hospital as an indicator of process quality; and inpatient mortality adjusted for patient characteristics as an indicator of outcome quality. We found regional variation to be greatest in SU density while variation in the other indicators was generally less pronounced. A positive trend of decreasing regional variation was found for the share of patients treated in a SU hospital, whereas the extent of regional variation in SU density and mortality remained largely stable. The states’ performances in relation to each other was also largely stable over time, indicating variations between states have a strong systematic component to them. Regarding state performances across indicators, some similarities in regional variation are seen between SU accessibility, treatment in SU hospitals, and mortality, while some states also show varying patterns of opposite directions.

### Access

Results for SU density show that the number of SU seems to stagnate since about 2017 at around 500 in Germany, which would be the first time since a separate remuneration for SU care was introduced in 2006 [24]. When only accounting for certified SU, as done in other studies [33], numbers reduce to about 350 SU in Germany, compared to the 500 SU identified in this study via a minimum of ten SU procedure codes per year [34]. Reimbursement of those SU treatments requires the provision of certain structural and process features, such as personnel staffing, monitoring, diagnostics, and therapy. Therefore, treatments according to SU procedure codes provide a good indicator for high quality stroke care, although the requirements are less strict compared to the certification process. Since certification of SU is voluntary, considering certified SU only would likely lead to an underestimation of adequately equipped sites. To avoid misclassification of hospitals as a ‘SU hospital’ due to coding errors we have set a pragmatic threshold of ten SU treatments per year. Increasing the threshold to 50 cases per year for a more conservative estimate results in about 460 SU (see Appendix Tab. A0-2). Regarding regional variation in SU density, higher density in rural areas can most likely be explained by larger units in bigger compared to smaller cities, which might also partly explain the comparatively large differences between the federal states. No other studies could be found assessing variations in SU density on a regional level.

The same is true for indicator 2, geographical accessibility of SU care. Since the vast majority of the German population could reach a SU hospital within 30 min by car, geographical accessibility can be considered as good (Germany: 95%, urban regions: 99%, rural regions: 91%). Regional variation between the federal states is also rather small, indicating a good distribution of SU hospitals. An exception applies to one state, where only two thirds of the population could reach a SU within 30 min by car. This state also serves as an example why it is important to assess both sub dimensions of access to care, as provider density can be high in sparsely populated areas due to low population (and stroke) figures, while distances between providers are large and thus geographical accessibility is low (and vice versa in densely populated regions).

### Quality

While no studies on regional variation in stroke care were found explicitly addressing access to care the way it was conceptualized in this study, many studies report measures of process quality or outcome quality, like provision of SU care, of thrombolysis, or stroke mortality. For instance, a systematic review of urban-rural differences in acute stroke care found evidence for lower process quality (e.g., frequency of SU care or thrombolysis) in rural areas, but differences seemed to diminish when controlling for hospital characteristics [35]. This unwarranted variation was (to a small extend) also found in Germany regarding the share of cases treated in hospitals with a SU, which was slightly higher in urban compared to rural regions in 2020, but with a stronger increase in rural regions in recent years. Similarly, a general positive trend was also seen in all federal states, and the variation between the states further decreased. In combination with the positive trend in SU density, this seems to indicate that SU care has been still rising until recently in Germany ever since its separate remuneration was introduced in 2006 [24], and that this development was initially slower in rural areas but differences have almost been dissolved.

For inpatient mortality in Germany as a whole, the decline between 2005–2010 from 11.9% to 9.5% reported in another study [24] seemed to first have continued but then stopped at around 8.5% in more recent years and rose to 9.0% in 2020. In terms of regional variation, risk-adjusted mortality rates were lower in rural compared to urban regions on a small but statistically significant level in all years. This is in contrast to a systematic review which found that some studies reported no difference in risk-adjusted stroke mortality, while others reported higher mortality in rural areas [35]. However, one should note that the assignment of inpatient cases to the respective regions in the mortality analysis of the present study was based on hospital locations. For the federal states, adjusted mortality varied around ±17% from the value for Germany as a whole. Significant levels of unwarranted regional variation in stroke mortality have also been found in health care atlases and in other single studies, e.g., [36–40], in one of which they could be largely ascribed to hospital characteristics [37]. However, a direct comparison of results is difficult due to methodological differences like available data or statistical computations used, e.g., factors included in risk-adjustments. A study on Germany covering years 1998–2015 found higher age-sex-standardized mortality in the eastern states formerly part of the German Democratic Republic, but differences were negligible at the end of the observation period [41]. In the present study additionally adjusting for comorbidities, mortality rates were above average in four of those six states in 2014 and in five of six in 2020, compared to two (2014) and four (2020) of the twelve other states, respectively, indicating inequalities are still present and seem to be (mostly) not attributable to morbidity patterns.

### Cross-indicator performance

Next to regional variation and trends for each indicator separately, the results also show patterns of health care performance in the federal states across indicators. While both access indicators are complementary to each other, i.e., provider availability and geographical accessibility both need to be sufficient to provide good access to care but they are not necessarily a consequence of each other, they are a prerequisite for treatments in SU hospitals, which itself is related to inpatient mortality. This is also reflected in the performance patterns between the states, which show similarities between geographical accessibility, frequency of SU treatments, and mortality (maps in Fig. 3). However, some states also deviate strongly from this expected pattern. For instance, one state in the north-east of the country with the by far lowest geographical accessibility of SU hospitals performs above average in the treatment in SU hospitals. Similarly, the state with the lowest inpatient mortality in both 2014 and 2020 clearly performs below average in the treatment in SU hospitals in both years. Such results might partly reflect methodological limitations of the indicators (e.g., transportation of stroke patients is also done by helicopter in Germany), but they nevertheless can represent a particular potential for identifying areas for further improvements in care provision. Yet, interrelations or performance patterns between indicators in regional variations seem to have been little studied so far (for an example, see [42]).

Another noteworthy result at the state level is a particular development in one small city-state in the north-west. In 2020, its number of inpatient stroke cases decreased by 42%, leading to a sharp increase in SU density, in contrast to the other states. In parallel, the share of inpatient cases treated in a SU hospital decreased from 88% in 2018 and 2019, respectively, to 74% in 2020, and inpatient mortality increased from 9.8% in 2018 to 11.5% in 2019 and 13.3% in 2020 . It remains unclear what produced these pronounced discrepancies and to what extent they might be influenced by the Covid-19 pandemic (in contrast to the other states). This should be further investigated, including developments in more recent years, to account for the caused health impacts.

### Implications for policy and further research

The findings also suggest possible policy measures for further improving stroke care in the regions. Although geographical accessibility of SU care was generally good, the map of travel times on the municipal level revealed some regional gaps, especially close to borders between the states. Since hospital planning is within the responsibility of the federal states, they might take measures here, especially since the scope to which the states engage in hospital planning activities differs. Other countries’ experiences show that regional planning of acute stroke care including transport times can improve access to high-quality care [43]. When it comes to allocation of patients to appropriate care, in this case SU, Germany already showed good results overall. However, there were also federal states in which both good availability and good geographical accessibility of SU care was not reflected accordingly in the share of cases treated in hospitals with a SU. In this case, measures like regional clinical pathways have been found to produce improvements [44]. In many studies assessing variation in stroke care, specialization was found to be a major driver of variation, e.g., between university and community hospitals [45, 46]. Consequently, studies found positive effects on quality for centralization of acute stroke care in metropolitan areas [47]. However, effects on access to care, especially in rural areas, must be considered when centralizing care. In Germany, further measures to improve quality are currently being developed as part of a hospital reform. Among other changes in financing mechanisms, remuneration of services will be more strongly dependent on adequate structures and equipment within hospitals in the future, and public reporting of quality measures is intended to increase a quality-based competition [48, 49].

However, this study included only four indicators covering the two health system dimensions access and quality, while health care atlases often focus on utilization, costs, and increasingly on quality (e.g., [50–53]). Further broadening that scope to best cover the care pathway or, in a broader sense, “system pathway” and evaluating performance patterns could provide additional evidence on mechanisms of regional variation and thus on which areas of care provision to improve, since research on addressing unwarranted variation is scarce, so far [4, 6, 54]. For stroke care, this might include more detailed indicators of process quality, additionally covering rehabilitation and prevention next to acute care, but also other health system dimensions like population health outcomes, patient-centredness, or efficiency.

### Strengths and limitations

While a major strength of this study is the combination of health system dimensions and indicators in measuring unwarranted regional variation, the employed data and indicator operationalizations do not come without limitations. This is especially true for SU density, because capacities of SU vary by their number of beds, which were not available for non-certified SU. However, considering certified SU only, as done in other studies, leads to a significant underestimation, as shown. A second limitation concerns the separation of unwarranted from warranted variation, which is difficult to accomplish both on a conceptual and on a measurement level [6, 55]. The routine data used in this study includes no information on patient preferences and also covers not all clinically relevant patient characteristics, e.g., physical factors not coded as secondary diagnoses in DRG data). Therefore, it is difficult to determine how much of the measured variation can be attributed to health system performance alone.

## Conclusions

The aim of this study was to assess regional variation in access to and quality of acute stroke care in Germany based on the results of Germany’s HSPA pilot report. Access to acute stroke care was found to be generally good. While statements about availability of SU care are limited since only SU density could be assessed instead of SU bed density, geographical accessibility is very high and regional variation is rather low. Still, a look at travel times on the municipal level revealed some regions where SU are lacking. Allocation of stroke patients to hospitals with a SU seems also to be working well and continued to improve, leading to a decreasing and generally low regional variation. Decreasing trends were not seen any more for inpatient mortality, in contrast to earlier studies, but regional variation was overall on a rather low level. It could also be shown that the indicators measured in this study, including their regional variation, were not particularly affected by the start of the Covid-19 pandemic in 2020, except for a mild increase in inpatient mortality. However, the results revealed some developments especially in one federal state that need further inspection.

Despite good overall results, the federal states might use their hospital planning competencies to close the remaining gaps in SU availability and geographical accessibility. Conversely, states with good access but poor results for patient allocation and mortality should further explore the underlying reasons and might install additional measures to improve care.

Overall, this study showed that combining different complementary measures of health care performance on a regional level creates further insights in variation and patterns of care which helps to better understand overall performance and to identify areas for improvements. Therefore, efforts in Health System Performance Assessment and in measuring unwarranted regional variation should be more strongly combined in the future.

### Supplementary Information

Supplementary Material 1.

## Data Availability

The data that support the findings of this study are available from the Research Data Centre of the Federal Statistical Office (German Diagnosis Related Groups statistics) but restrictions apply to the availability of these data, which were used under license for the current study, and so are not publicly available [23]. Data on hospital quality reports are available from the German Federal Joint Committee [21].

## Footnotes

1 For instance, individual case characteristics might prevent patients from receiving SU care. Additionally, procedure codes for SU care can only be used for remuneration purposes if patients received treatments at least for 24h, which leads to an underreporting in cases where patients decease during that time.

2 Comorbidities include heart failure/cardiomyopathy, chronic ischaemic heart disease, hypertension, valvular heart diseases, atherosclerosis of the extremities, COPD/asthma, liver disease, severe kidney disease or chronic renal failure, diabetes mellitus, obesity, cachexia/malnutrition, coagulation disorder, malignant neoplasm, Covid-19 confirmed (see Appendix Tab. A4–6 for details)
