# Supplementary material for: Regional variation in access to and quality of acute stroke care: results of Germany’s Health System Performance Assessment pilot, 2014–2020
Source: Res Health Serv Reg. 2024 Jul 2;3:9. doi: 10.1007/s43999-024-00045-x (PMC11281753; doi:10.1007/s43999-024-00045-x)
Supplement: Supplementary file 1 — Supplementary Material 1. [file 43999_2024_45_MOESM1_ESM.doc]

**Appendix**

Content

[***Supplementary tables A0: Descriptive results for number of stroke units (SU) and inpatient stroke cases*** 2](#__RefHeading___Toc167458618)

[*Tab. A0-1 Number of SU, defined as hospitals with ten or more SU procedure codes per year in quality reports (Germany, 16 federal states, urban and rural regions, 2014-2020)* 2](#__RefHeading___Toc167458619)

[*Tab. A0-2 Number of SU, alternatively defined as hospitals with fifty or more SU procedure codes per year in quality reports (Germany, 16 federal states, urban and rural regions, 2014-2020)* 2](#__RefHeading___Toc167458620)

[*Tab. A0-3 Number of inpatient stroke cases per year (Germany, 16 federal states, urban and rural regions, 2014-2020)* 3](#__RefHeading___Toc167458621)

[***Supplementary tables A1: Indicator 1: Stroke unit (SU) density per 1,000 inpatient stroke cases*** 4](#__RefHeading___Toc167458622)

[*Tab. A1-1 SU density per 1,000 inpatient stroke cases (Germany, 16 federal states, urban and rural regions, 2014-2020)* 4](#__RefHeading___Toc167458623)

[*Tab. A1-2 SU density per 1,000 inpatient stroke cases, relative deviation from values for Germany as a whole in % (16 federal states, urban and rural regions, 2014-2020)* 4](#__RefHeading___Toc167458624)

[***Supplementary tables A2: Indicator 2: Share of the population reaching a stroke unit (SU) within 30min by car (available for 2020 only)*** 5](#__RefHeading___Toc167458625)

[*Tab. A2-1 Share of the population reaching a SU within 30min by car in % (Germany, 16 federal states, urban and rural regions, 2020)* 5](#__RefHeading___Toc167458626)

[*Tab. A2-2 Share of the population reaching a SU within 30min by car, relative deviation from values for Germany as a whole in % (16 federal states, urban and rural regions, 2020)* 5](#__RefHeading___Toc167458627)

[***Supplementary tables A3: Indicator 3: Share of inpatient stroke cases treated in a hospital with a stroke unit (SU)*** 6](#__RefHeading___Toc167458628)

[*Tab. A3-1 Share of inpatient stroke cases treated in a hospital with a SU in % (Germany, 16 federal states, urban and rural regions, 2014-2020)* 6](#__RefHeading___Toc167458629)

[*Tab. A3-2 Share of inpatient stroke cases treated in a hospital with a SU, relative deviation from values for Germany as a whole in % (16 federal states, urban and rural regions, 2014-2020)* 6](#__RefHeading___Toc167458630)

[***Supplementary tables A4: Indicator 4: Inpatient mortality*** 7](#__RefHeading___Toc167458631)

[*Tab. A4-1 Crude inpatient mortality rates in % (Germany, 16 federal states, urban and rural regions, 2014-2020)* 7](#__RefHeading___Toc167458632)

[*Tab. A4-2 Adjusted inpatient mortality rates in %, based on regression models for each calendar year separately (16 federal states, urban and rural regions, 2014-2020)* 8](#__RefHeading___Toc167458633)

[*Tab. A4-3 Standardized mortality ratios (SMR) and 95% confidence intervals (CI), based on regression models for each calendar year separately (16 federal states, urban and rural regions, 2014-2020)* 8](#__RefHeading___Toc167458634)

[*Tab. A4-4 Adjusted inpatient mortality rates in %, based on regression models for all years combined (16 federal states, urban and rural regions, 2014-2020)* 9](#__RefHeading___Toc167458635)

[*Tab. A4-5 Standardized mortality ratios (SMR) and 95% confidence intervals (CI), based on regression models for all years combined (16 federal states, urban and rural regions, 2014-2020)* 9](#__RefHeading___Toc167458636)

[*Tab. A4-6 Descriptives and results for control variables of regression models on inpatient mortality (models for each calendar year separately and all years combined, respectively)* 10](#__RefHeading___Toc167458637)

***Supplementary tables A0: Descriptive results for number of stroke units (SU) and inpatient stroke cases***

*Tab. A0-1 Number of SU, defined as hospitals with ten or more SU procedure codes per year in quality reports (Germany, 16 federal states, urban and rural regions, 2014-2020)*

|  | 2014 | 2015 | 2016 | 2017 | 2018 | 2019 | 2020 |
| --- | --- | --- | --- | --- | --- | --- | --- |
| GER | 477 | 489 | 500 | 506 | 503 | 500 | 485 |
| BB | 21 | 22 | 23 | 24 | 25 | 26 | 25 |
| BE | 14 | 16 | 17 | 15 | 15 | 14 | 14 |
| BW | 52 | 50 | 54 | 55 | 53 | 54 | 53 |
| BY | 83 | 89 | 90 | 90 | 88 | 87 | 83 |
| HB | 2 | 2 | 3 | 3 | 3 | 3 | 3 |
| HE | 32 | 33 | 32 | 32 | 33 | 34 | 33 |
| HH | 11 | 11 | 11 | 10 | 11 | 10 | 10 |
| MV | 11 | 10 | 10 | 10 | 11 | 11 | 11 |
| NI | 40 | 41 | 42 | 44 | 43 | 47 | 44 |
| NW | 95 | 95 | 97 | 95 | 92 | 88 | 85 |
| RP | 27 | 27 | 27 | 30 | 31 | 31 | 28 |
| SH | 19 | 19 | 21 | 19 | 19 | 20 | 19 |
| SL | 10 | 10 | 10 | 10 | 10 | 10 | 10 |
| SN | 24 | 24 | 26 | 28 | 28 | 27 | 29 |
| ST | 18 | 20 | 18 | 19 | 20 | 18 | 18 |
| TH | 18 | 20 | 19 | 22 | 21 | 20 | 20 |
| Urban | 202 | 205 | 212 | 207 | 208 | 201 | 198 |
| Rural | 275 | 284 | 288 | 299 | 295 | 299 | 287 |

*Tab. A0-2 Number of SU, alternatively defined as hospitals with fifty or more SU procedure codes per year in quality reports (Germany, 16 federal states, urban and rural regions, 2014-2020)*

|  | 2014 | 2015 | 2016 | 2017 | 2018 | 2019 | 2020 |
| --- | --- | --- | --- | --- | --- | --- | --- |
| GER | 441 | 451 | 455 | 466 | 462 | 463 | 460 |
| BB | 20 | 19 | 20 | 20 | 19 | 21 | 20 |
| BE | 13 | 15 | 17 | 15 | 14 | 14 | 14 |
| BW | 47 | 47 | 50 | 53 | 51 | 51 | 52 |
| BY | 73 | 80 | 80 | 81 | 81 | 80 | 77 |
| HB | 2 | 2 | 2 | 3 | 3 | 3 | 3 |
| HE | 30 | 30 | 31 | 31 | 32 | 32 | 32 |
| HH | 11 | 11 | 10 | 10 | 11 | 10 | 10 |
| MV | 10 | 9 | 9 | 9 | 11 | 11 | 10 |
| NI | 37 | 37 | 38 | 40 | 41 | 42 | 43 |
| NW | 84 | 86 | 85 | 84 | 81 | 82 | 83 |
| RP | 26 | 26 | 25 | 27 | 28 | 27 | 25 |
| SH | 19 | 19 | 18 | 18 | 18 | 18 | 19 |
| SL | 10 | 10 | 10 | 10 | 10 | 10 | 10 |
| SN | 24 | 24 | 25 | 27 | 27 | 25 | 27 |
| ST | 17 | 17 | 18 | 18 | 16 | 17 | 15 |
| TH | 18 | 19 | 17 | 20 | 19 | 20 | 20 |
| Urban | 189 | 195 | 196 | 195 | 192 | 193 | 191 |
| Rural | 252 | 256 | 259 | 271 | 70 | 270 | 269 |

*Tab. A0-3 Number of inpatient stroke cases per year (Germany, 16 federal states, urban and rural regions, 2014-2020)*

|  | 2014 | 2015 | 2016 | 2017 | 2018 | 2019 | 2020 |
| --- | --- | --- | --- | --- | --- | --- | --- |
| GER | 248,458 | 253,501 | 257,433 | 256,594 | 252,902 | 252,843 | 238,384 |
| BB | 7,962 | 7,859 | 7,916 | 7,983 | 7,792 | 7,604 | 7,204 |
| BE | 9,929 | 10,273 | 10,456 | 10,707 | 10,334 | 10,098 | 9,698 |
| BW | 30,084 | 30,703 | 31,658 | 31,552 | 31,284 | 31,194 | 29,528 |
| BY | 37,717 | 38,372 | 39,096 | 39,131 | 38,464 | 38,598 | 36,174 |
| HB | 3,121 | 3,297 | 3,352 | 3,500 | 3,412 | 3,353 | 1,921 |
| HE | 16,869 | 17,363 | 17,299 | 17,580 | 17,564 | 17,321 | 16,651 |
| HH | 6,644 | 6,872 | 6,890 | 6,734 | 6,762 | 6,745 | 6,399 |
| MV | 4,992 | 5,072 | 5,192 | 5,048 | 5,170 | 5,651 | 5,314 |
| NI | 23,949 | 24,334 | 24,727 | 24,606 | 24,200 | 23,968 | 23,193 |
| NW | 53,606 | 54,747 | 55,836 | 55,272 | 54,877 | 54,626 | 51,854 |
| RP | 12,401 | 12,438 | 12,414 | 12,128 | 11,917 | 11,959 | 11,208 |
| SH | 8,000 | 8,215 | 8,556 | 8,423 | 8,354 | 8,392 | 7,755 |
| SL | 4,338 | 4,381 | 4,291 | 4,309 | 4,171 | 4,232 | 4,016 |
| SN | 13,102 | 13,732 | 13,535 | 13,520 | 13,040 | 13,496 | 12,410 |
| ST | 8,343 | 8,183 | 8,351 | 8,270 | 7,919 | 8,036 | 7,746 |
| TH | 7,401 | 7,660 | 7,864 | 7,831 | 7,642 | 7,570 | 7,313 |
| Urban | 114,579 | 117,354 | 119,507 | 119,837 | 117,941 | 117,560 | 109,833 |
| Rural | 133,879 | 136,147 | 137,926 | 136,757 | 134,961 | 135,283 | 128,551 |

***Supplementary tables A1: Indicator 1: Stroke unit (SU) density per 1,000 inpatient stroke cases***

*Tab. A1-1 SU density per 1,000 inpatient stroke cases (Germany, 16 federal states, urban and rural regions, 2014-2020)*

|  | 2014 | 2015 | 2016 | 2017 | 2018 | 2019 | 2020 |
| --- | --- | --- | --- | --- | --- | --- | --- |
| GER | 1.92 | 1.93 | 1.94 | 1.97 | 1.99 | 1.98 | 2.03 |
| BB | 2.64 | 2.80 | 2.91 | 3.01 | 3.21 | 3.42 | 3.47 |
| BE | 1.41 | 1.56 | 1.63 | 1.40 | 1.45 | 1.39 | 1.44 |
| BW | 1.73 | 1.63 | 1.71 | 1.74 | 1.69 | 1.73 | 1.79 |
| BY | 2.20 | 2.32 | 2.30 | 2.30 | 2.29 | 2.25 | 2.29 |
| HB | 0.64 | 0.61 | 0.89 | 0.86 | 0.88 | 0.89 | 1.56 |
| HE | 1.90 | 1.90 | 1.85 | 1.82 | 1.88 | 1.96 | 1.98 |
| HH | 1.66 | 1.60 | 1.60 | 1.49 | 1.63 | 1.48 | 1.56 |
| MV | 2.20 | 1.97 | 1.93 | 1.98 | 2.13 | 1.95 | 2.07 |
| NI | 1.67 | 1.68 | 1.70 | 1.79 | 1.78 | 1.96 | 1.90 |
| NW | 1.77 | 1.74 | 1.74 | 1.72 | 1.68 | 1.61 | 1.64 |
| RP | 2.18 | 2.17 | 2.17 | 2.47 | 2.60 | 2.59 | 2.50 |
| SH | 2.38 | 2.31 | 2.45 | 2.26 | 2.27 | 2.38 | 2.45 |
| SL | 2.31 | 2.28 | 2.33 | 2.32 | 2.40 | 2.36 | 2.49 |
| SN | 1.83 | 1.75 | 1.92 | 2.07 | 2.15 | 2.00 | 2.34 |
| ST | 2.16 | 2.44 | 2.16 | 2.30 | 2.53 | 2.24 | 2.32 |
| TH | 2.43 | 2.61 | 2.42 | 2.81 | 2.75 | 2.64 | 2.73 |
| Urban | 1.76 | 1.75 | 1.77 | 1.73 | 1.76 | 1.71 | 1.80 |
| Rural | 2.05 | 2.09 | 2.09 | 2.19 | 2.19 | 2.21 | 2.23 |

*Tab. A1-2 SU density per 1,000 inpatient stroke cases, relative deviation from values for Germany as a whole in % (16 federal states, urban and rural regions, 2014-2020)*

|  | 2014 | 2015 | 2016 | 2017 | 2018 | 2019 | 2020 |
| --- | --- | --- | --- | --- | --- | --- | --- |
| BB | +37.4 | +45.1 | +49.6 | +52.5 | +61.3 | +72.9 | +70.6 |
| BE | -26.6 | -19.3 | -16.3 | -29.0 | -27.0 | -29.9 | -29.0 |
| BW | -10.0 | -15.6 | -12.2 | -11.6 | -14.8 | -12.5 | -11.8 |
| BY | +14.6 | +20.2 | +18.5 | +16.6 | +15.0 | +14.0 | +12.8 |
| HB | -66.6 | -68.6 | -53.9 | -56.5 | -55.8 | -54.8 | -23.2 |
| HE | -1.2 | -1.5 | -4.8 | -7.7 | -5.5 | -0.7 | -2.6 |
| HH | -13.8 | -17.0 | -17.8 | -24.7 | -18.2 | -25.0 | -23.2 |
| MV | +14.8 | +2.2 | -0.8 | +0.5 | +7.0 | -1.6 | +1.7 |
| NI | -13.0 | -12.7 | -12.5 | -9.3 | -10.7 | -0.8 | -6.8 |
| NW | -7.7 | -10.0 | -10.6 | -12.8 | -15.7 | -18.5 | -19.4 |
| RP | +13.4 | +12.5 | +12.0 | +25.4 | +30.8 | +31.1 | +22.8 |
| SH | +23.7 | +19.9 | +26.4 | +14.4 | +14.4 | +20.5 | +20.4 |
| SL | +20.1 | +18.3 | +20.0 | +17.7 | +20.5 | +19.5 | +22.4 |
| SN | -4.6 | -9.4 | -1.1 | +5.0 | +8.0 | +1.2 | +14.9 |
| ST | +12.4 | +26.7 | +11.0 | +16.5 | +27.0 | +13.3 | +14.2 |
| TH | +26.7 | +35.4 | +24.4 | +42.5 | +38.2 | +33.6 | +34.4 |
| Urban | -8.2 | -9.4 | -8.7 | -12.4 | -11.3 | -13.5 | -11.4 |
| Rural | +7.0 | +8.1 | +7.5 | +10.9 | +9.9 | +11.8 | +9.7 |

***Supplementary tables A2: Indicator 2: Share of the population reaching a stroke unit (SU) within 30min by car (available for 2020 only)***

*Tab. A2-1 Share of the population reaching a SU within 30min by car in % (Germany, 16 federal states, urban and rural regions, 2020)*

|  | 2020 |
| --- | --- |
| GER | 94.71 |
| BB | 90.27 |
| BE | 100.00 |
| BW | 97.96 |
| BY | 95.83 |
| HB | 100.00 |
| HE | 95.52 |
| HH | 100.00 |
| MV | 68.29 |
| NI | 90.62 |
| NW | 97.14 |
| RP | 91.86 |
| SH | 94.43 |
| SL | 99.07 |
| SN | 89.90 |
| ST | 88.25 |
| TH | 94.07 |
| Urban | 99.35 |
| Rural | 90.51 |

*Tab. A2-2 Share of* the population reaching a SU within 30min by car, relative deviation from values for Germany as a whole in % (16 federal states, urban and rural regions, 2020)

|  | 2020 |
| --- | --- |
| BB | -4.7 |
| BE | +5.6 |
| BW | +3.4 |
| BY | +1.2 |
| HB | +5.6 |
| HE | +0.9 |
| HH | +5.6 |
| MV | -27.9 |
| NI | -4.3 |
| NW | +2.6 |
| RP | -3.0 |
| SH | -0.3 |
| SL | +4.6 |
| SN | -5.1 |
| ST | -6.8 |
| TH | -0.7 |
| Urban | +4.9 |
| Rural | -4.4 |

***Supplementary tables A3: Indicator 3: Share of inpatient stroke cases treated in a hospital with a stroke unit (SU)***

*Tab. A3-1 Share of inpatient stroke cases treated in a hospital with a SU in % (Germany, 16 federal states, urban and rural regions, 2014-2020)*

|  | 2014 | 2015 | 2016 | 2017 | 2018 | 2019 | 2020 |
| --- | --- | --- | --- | --- | --- | --- | --- |
| GER | 86.89 | 87.80 | 90.19 | 91.59 | 92.11 | 93.12 | 92.94 |
| BB | 91.81 | 92.53 | 89.99 | 93.69 | 93.71 | 94.74 | 94.11 |
| BE | 95.39 | 95.86 | 96.47 | 96.93 | 96.97 | 97.66 | 97.96 |
| BW | 88.54 | 87.00 | 93.43 | 94.76 | 95.24 | 96.97 | 97.24 |
| BY | 87.98 | 90.49 | 90.53 | 91.37 | 90.95 | 92.14 | 92.01 |
| HB | 75.62 | 76.98 | 83.83 | 86.89 | 87.63 | 87.62 | 74.28 |
| HE | 92.54 | 91.69 | 92.77 | 92.67 | 92.68 | 94.48 | 94.82 |
| HH | 97.23 | 97.32 | 97.71 | 97.06 | 97.43 | 97.39 | 97.52 |
| MV | 86.96 | 86.71 | 87.96 | 90.10 | 93.85 | 94.85 | 96.26 |
| NI | 81.71 | 85.14 | 88.44 | 90.73 | 91.91 | 93.52 | 92.82 |
| NW | 86.78 | 87.14 | 89.72 | 91.17 | 91.91 | 92.81 | 93.64 |
| RP | 81.32 | 81.75 | 85.61 | 88.95 | 90.82 | 90.23 | 88.85 |
| SH | 96.12 | 96.07 | 96.75 | 96.46 | 97.34 | 97.37 | 95.62 |
| SL | 95.85 | 96.53 | 96.81 | 97.54 | 97.82 | 98.16 | 98.73 |
| SN | 70.79 | 72.29 | 78.32 | 80.73 | 81.02 | 82.28 | 79.54 |
| ST | 84.27 | 87.55 | 88.04 | 89.71 | 89.01 | 89.83 | 88.45 |
| TH | 83.48 | 86.96 | 87.30 | 88.39 | 89.06 | 89.58 | 89.74 |
| Urban | 90.09 | 90.39 | 93.13 | 93.80 | 94.10 | 94.95 | 94.65 |
| Rural | 84.14 | 85.56 | 87.64 | 89.65 | 90.37 | 91.53 | 91.47 |

*Tab. A3-2 Share of inpatient stroke cases treated in a hospital with a SU, relative deviation from values for Germany as a whole in % (16 federal states, urban and rural regions, 2014-2020)*

|  | 2014 | 2015 | 2016 | 2017 | 2018 | 2019 | 2020 |
| --- | --- | --- | --- | --- | --- | --- | --- |
| BB | +5.7 | +5.4 | -0.2 | +2.3 | +1.7 | +1.7 | +1.3 |
| BE | +9.8 | +9.2 | +7.0 | +5.8 | +5.3 | +4.9 | +5.4 |
| BW | +1.9 | -0.9 | +3.6 | +3.5 | +3.4 | +4.1 | +4.6 |
| BY | +1.3 | +3.1 | +0.4 | -0.2 | -1.3 | -1.0 | -1.0 |
| HB | -13.0 | -12.3 | -7.1 | -5.1 | -4.9 | -5.9 | -20.1 |
| HE | +6.5 | +4.4 | +2.9 | +1.2 | +0.6 | +1.5 | +2.0 |
| HH | +11.9 | +10.8 | +8.3 | +6.0 | +5.8 | +4.6 | +4.9 |
| MV | +0.1 | -1.2 | -2.5 | -1.6 | +1.9 | +1.9 | +3.6 |
| NI | -6.0 | -3.0 | -1.9 | -0.9 | -0.2 | +0.4 | -0.1 |
| NW | -0.1 | -0.7 | -0.5 | -0.5 | -0.2 | -0.3 | +0.8 |
| RP | -6.4 | -6.9 | -5.1 | -2.9 | -1.4 | -3.1 | -4.4 |
| SH | +10.6 | +9.4 | +7.3 | +5.3 | +5.7 | +4.6 | +2.9 |
| SL | +10.3 | +9.9 | +7.3 | +6.5 | +6.2 | +5.4 | +6.2 |
| SN | -18.5 | -17.7 | -13.2 | -11.9 | -12.0 | -11.6 | -14.4 |
| ST | -3.0 | -0.3 | -2.4 | -2.1 | -3.4 | -3.5 | -4.8 |
| TH | -3.9 | -1.0 | -3.2 | -3.5 | -3.3 | -3.8 | -3.4 |
| Urban | +3.7 | +3.0 | +3.3 | +2.4 | 2.2 | +2.0 | +1.8 |
| Rural | -3.2 | -2.5 | -2.8 | -2.1 | -1.9 | -1.7 | -1.6 |

***Supplementary tables A4: Indicator 4: Inpatient mortality***

*Tab. A4-1 Crude inpatient mortality rates in % (Germany, 16 federal states, urban and rural regions, 2014-2020)*

|  | 2014 | 2015 | 2016 | 2017 | 2018 | 2019 | 2020 |
| --- | --- | --- | --- | --- | --- | --- | --- |
| GER | 8.55 | 8.67 | 8.37 | 8.45 | 8.45 | 8.56 | 8.97 |
| BB | 8.55 | 9.35 | 8.46 | 8.09 | 7.47 | 7.56 | 8.19 |
| BE | 8.58 | 8.24 | 9.10 | 8.39 | 9.06 | 8.90 | 9.86 |
| BW | 8.36 | 8.63 | 8.43 | 8.26 | 8.49 | 8.75 | 9.14 |
| BY | 8.35 | 8.51 | 7.97 | 8.56 | 7.83 | 8.19 | 8.92 |
| HB | 8.20 | 9.65 | 9.55 | 9.40 | 9.53 | 10.89 | 14.32 |
| HE | 7.71 | 7.95 | 7.54 | 8.07 | 8.03 | 7.95 | 8.46 |
| HH | 7.65 | 7.10 | 6.81 | 7.96 | 8.16 | 8.30 | 8.70 |
| MV | 8.11 | 8.32 | 8.22 | 8.54 | 9.05 | 9.73 | 9.20 |
| NI | 8.49 | 8.25 | 8.22 | 8.16 | 8.12 | 7.79 | 8.33 |
| NW | 9.09 | 9.15 | 8.85 | 8.62 | 8.70 | 9.00 | 9.19 |
| RP | 7.81 | 8.03 | 7.69 | 7.77 | 7.80 | 7.87 | 8.01 |
| SH | 8.61 | 8.06 | 7.30 | 7.72 | 8.51 | 8.32 | 8.10 |
| SL | 7.47 | 7.76 | 7.20 | 8.68 | 8.15 | 8.58 | 9.06 |
| SN | 10.46 | 10.80 | 9.87 | 9.84 | 9.75 | 9.60 | 9.98 |
| ST | 8.34 | 8.53 | 8.69 | 8.92 | 9.22 | 8.82 | 8.79 |
| TH | 8.36 | 8.98 | 8.80 | 8.54 | 9.21 | 8.32 | 9.08 |
| Urban | 8.76 | 8.86 | 8.64 | 8.64 | 8.75 | 8.90 | 9.34 |
| Rural | 8.37 | 8.51 | 8.13 | 8.29 | 8.19 | 8.26 | 8.65 |

*Tab. A4-2 Adjusted inpatient mortality rates in %, based on regression models for each calendar year separately (16 federal states, urban and rural regions, 2014-2020)*

|  | 2014 | 2015 | 2016 | 2017 | 2018 | 2019 | 2020 |
| --- | --- | --- | --- | --- | --- | --- | --- |
| BB | 9.14 | 9.98 | 8.83 | 8.57 | 7.96 | 7.82 | 8.68 |
| BE | 8.31 | 8.24 | 8.86 | 8.32 | 8.75 | 8.91 | 9.52 |
| BW | 8.18 | 8.39 | 8.15 | 8.09 | 8.36 | 8.52 | 8.98 |
| BY | 8.04 | 8.22 | 7.77 | 8.32 | 7.57 | 7.92 | 8.56 |
| HB | 8.79 | 10.00 | 9.59 | 9.58 | 9.85 | 11.52 | 13.33 |
| HE | 7.87 | 8.11 | 7.85 | 8.28 | 8.42 | 8.15 | 8.62 |
| HH | 8.23 | 7.46 | 7.03 | 7.91 | 7.92 | 8.29 | 8.72 |
| MV | 8.77 | 8.71 | 8.88 | 8.88 | 9.33 | 10.36 | 9.58 |
| NI | 8.48 | 8.39 | 8.35 | 8.25 | 8.30 | 8.02 | 8.54 |
| NW | 9.18 | 9.14 | 8.87 | 8.66 | 8.75 | 8.99 | 9.25 |
| RP | 7.87 | 8.36 | 7.91 | 8.09 | 8.04 | 8.13 | 8.22 |
| SH | 8.33 | 8.01 | 7.48 | 7.78 | 8.60 | 8.39 | 8.49 |
| SL | 8.09 | 8.24 | 8.06 | 9.50 | 8.57 | 9.42 | 9.71 |
| SN | 9.75 | 10.13 | 9.23 | 9.29 | 9.26 | 9.11 | 9.42 |
| ST | 8.54 | 8.81 | 8.89 | 9.22 | 9.51 | 8.94 | 9.22 |
| TH | 9.12 | 9.34 | 8.82 | 8.40 | 9.11 | 8.44 | 9.36 |
| Urban | 8.81 | 8.88 | 8.63 | 8.64 | 8.70 | 8.87 | 9.24 |
| Rural | 8.33 | 8.50 | 8.14 | 8.29 | 8.23 | 8.28 | 8.73 |

Tab. A4-3 Standardized mortality ratios (SMR) and 95% confidence intervals (CI), based on regression models for each calendar year separately (16 federal states, urban and rural regions, 2014-2020)

|  | 2014 | 2015 | 2016 | 2017 | 2018 | 2019 | 2020 |
| --- | --- | --- | --- | --- | --- | --- | --- |
| BB | 1.07 (0.99;1.15) | 1.15 (1.07;1.24) | 1.05 (0.98;1.14) | 1.01 (0.94;1.10) | 0.94 (0.87;1.02) | **0.91 (0.84;0.99)** | 0.97 (0.89;1.05) |
| BE | 0.97 (0.91;1.04) | 0.95 (0.89;1.02) | 1.06 (0.99;1.13) | 0.98 (0.92;1.05) | 1.04 (0.97;1.10) | 1.04 (0.97;1.11) | **1.06 (1.00;1.13)** |
| BW | **0.96 (0.92;0.99)** | 0.97 (0.93;1.01) | 0.97 (0.94;1.01) | **0.96 (0.92;0.99)** | 0.99 (0.95;1.03) | 1.00 (0.96;1.03) | 1.00 (0.96;1.04) |
| BY | **0.94 (0.91;0.97)** | **0.95 (0.92;0.98)** | **0.93 (0.90;0.96)** | 0.98 (0.95;1.02) | **0.90 (0.86;0.93)** | **0.93 (0.89;0.96)** | **0.96 (0.92;0.99)** |
| HB | 1.03 (0.91;1.16) | **1.15 (1.03;1.29)** | **1.15 (1.02;1.28)** | **1.13 (1.01;1.26)** | **1.17 (1.04;1.30)** | **1.35 (1.21;1.49)** | **1.49 (1.32;1.67)** |
| HE | **0.92 (0.87;0.97)** | **0.94 (0.89;0.99)** | **0.94 (0.89;0.99)** | 0.98 (0.93;1.03) | 1.00 (0.94;1.05) | **0.95 (0.90;1.00)** | 0.96 (0.91;1.01) |
| HH | 0.96 (0.88;1.05) | **0.86 (0.78;0.94)** | **0.84 (0.77;0.92)** | 0.94 (0.86;1.02) | 0.94 (0.86;1.02) | 0.97 (0.89;1.05) | 0.97 (0.89;1.06) |
| MV | 1.03 (0.93;1.13) | 1.00 (0.91;1.10) | 1.06 (0.96;1.17) | 1.05 (0.95;1.15) | **1.10 (1.01;1.21)** | **1.21 (1.11;1.32)** | 1.07 (0.98;1.17) |
| NI | 0.99 (0.95;1.04) | 0.97 (0.93;1.01) | 1.00 (0.96;1.04) | 0.98 (0.93;1.02) | 0.98 (0.94;1.03) | **0.94 (0.89;0.98)** | **0.95 (0.91;1.00)** |
| NW | **1.07 (1.04;1.10)** | **1.05 (1.02;1.08)** | **1.06 (1.03;1.09)** | **1.02 (1.00;1.05)** | **1.04 (1.01;1.07)** | **1.05 (1.02;1.08)** | **1.03 (1.00;1.06)** |
| RP | **0.92 (0.86;0.98)** | 0.96 (0.91;1.03) | 0.95 (0.89;1.01) | 0.96 (0.90;1.02) | 0.95 (0.89;1.01) | 0.95 (0.89;1.01) | **0.92 (0.86;0.98)** |
| SH | 0.97 (0.90;1.05) | **0.92 (0.85;1.00)** | **0.89 (0.82;0.97)** | **0.92 (0.85;0.99)** | 1.02 (0.94;1.09) | 0.98 (0.91;1.06) | 0.95 (0.87;1.02) |
| SL | 0.95 (0.85;1.05) | 0.95 (0.85;1.06) | 0.96 (0.86;1.08) | **1.12 (1.01;1.24)** | 1.01 (0.91;1.13) | 1.10 (0.99;1.22) | 1.08 (0.97;1.20) |
| SN | **1.14 (1.08;1.20)** | **1.17 (1.11;1.23)** | **1.10 (1.04;1.16)** | **1.10 (1.04;1.16)** | **1.10 (1.04;1.16)** | **1.07 (1.01;1.12)** | 1.05 (0.99;1.11) |
| ST | 1.00 (0.93;1.08) | 1.02 (0.94;1.09) | 1.06 (0.99;1.14) | **1.09 (1.01;1.17)** | **1.13 (1.04;1.21)** | 1.05 (0.97;1.12) | 1.03 (0.95;1.11) |
| TH | 1.07 (0.98;1.15) | **1.08 (1.00;1.16)** | 1.05 (0.98;1.14) | 0.99 (0.92;1.07) | **1.08 (1.00;1.16)** | 0.99 (0.91;1.07) | 1.04 (0.97;1.13) |
| Urban | **1.03 (1.01;1.05)** | **1.02 (1.00;1.04)** | **1.03 (1.01;1.05)** | **1.02 (1.00;1.04)** | **1.03 (1.01;1.05)** | **1.04 (1.02;1.06)** | **1.03 (1.01;1.05)** |
| Rural | **0.97 (0.96;0.99)** | **0.98 (0.96;1.00)** | **0.97 (0.96;0.99)** | **0.98 (0.96;1.00)** | **0.97 (0.96;0.99)** | **0.97 (0.95;0.99)** | **0.97 (0.96;0.99)** |

*Statistically significant values in bold face.*

*Tab. A4-4 Adjusted inpatient mortality rates in %, based on regression models for all years combined (16 federal states, urban and rural regions, 2014-2020)*

|  | 2014 | 2015 | 2016 | 2017 | 2018 | 2019 | 2020 |
| --- | --- | --- | --- | --- | --- | --- | --- |
| BB | 9.19 | 9.89 | 9.03 | 8.72 | 8.08 | 7.82 | 8.21 |
| BE | 8.36 | 8.13 | 9.06 | 8.43 | 8.89 | 8.96 | 9.09 |
| BW | 8.18 | 8.28 | 8.34 | 8.21 | 8.48 | 8.54 | 8.61 |
| BY | 8.06 | 8.13 | 7.96 | 8.43 | 7.67 | 7.92 | 8.18 |
| HB | 8.80 | 9.85 | 9.81 | 9.71 | 9.99 | 11.53 | 12.84 |
| HE | 7.87 | 8.01 | 8.03 | 8.40 | 8.54 | 8.16 | 8.26 |
| HH | 8.25 | 7.35 | 7.18 | 8.02 | 8.04 | 8.32 | 8.35 |
| MV | 8.85 | 8.59 | 9.07 | 9.01 | 9.49 | 10.38 | 9.14 |
| NI | 8.52 | 8.30 | 8.56 | 8.36 | 8.40 | 8.02 | 8.15 |
| NW | 9.21 | 9.03 | 9.09 | 8.77 | 8.88 | 9.01 | 8.84 |
| RP | 7.89 | 8.28 | 8.11 | 8.20 | 8.14 | 8.12 | 7.85 |
| SH | 8.36 | 7.90 | 7.65 | 7.88 | 8.71 | 8.41 | 8.09 |
| SL | 8.07 | 8.13 | 8.25 | 9.65 | 8.70 | 9.45 | 9.25 |
| SN | 9.77 | 10.02 | 9.46 | 9.44 | 9.38 | 9.12 | 9.00 |
| ST | 8.58 | 8.71 | 9.09 | 9.35 | 9.65 | 8.94 | 8.78 |
| TH | 9.14 | 9.24 | 9.04 | 8.53 | 9.26 | 8.46 | 8.92 |
| Urban | 8.82 | 8.76 | 8.83 | 8.76 | 8.84 | 8.89 | 8.84 |
| Rural | 8.36 | 8.41 | 8.34 | 8.41 | 8.34 | 8.29 | 8.34 |

Tab. A4-5 Standardized mortality ratios (SMR) and 95% confidence intervals (CI), based on regression models for all years combined (16 federal states, urban and rural regions, 2014-2020)

|  | 2014 | 2015 | 2016 | 2017 | 2018 | 2019 | 2020 |
| --- | --- | --- | --- | --- | --- | --- | --- |
| BB | 1.07 (0.99;1.16) | **1.15 (1.07;1.24)** | 1.05 (0.98;1.14) | 1.02 (0.94;1.10) | 0.94 (0.87;1.02) | **0.91 (0.84;0.99)** | 0.96 (0.88;1.04) |
| BE | 0.98 (0.91;1.04) | 0.95 (0.89;1.01) | 1.06 (0.99;1.13) | 0.98 (0.92;1.05) | 1.04 (0.97;1.11) | 1.05 (0.98;1.12) | 1.06 (0.99;1.13) |
| BW | **0.95 (0.92;0.99)** | **0.97 (0.93;1.00)** | 0.97 (0.94;1.01) | **0.96 (0.92;0.99)** | 0.99 (0.95;1.03) | 1.00 (0.96;1.03) | 1.01 (0.97;1.04) |
| BY | **0.94 (0.91;0.97)** | **0.95 (0.92;0.98)** | **0.93 (0.90;0.96)** | 0.98 (0.95;1.02) | **0.90 (0.86;0.93)** | **0.92 (0.89;0.96)** | **0.95 (0.92;0.99)** |
| HB | 1.03 (0.91;1.16) | **1.15 (1.03;1.28)** | **1.14 (1.02;1.28)** | **1.13 (1.01;1.26)** | **1.17 (1.04;1.30)** | **1.34 (1.21;1.49)** | **1.50 (1.33;1.69)** |
| HE | **0.92 (0.87;0.97)** | **0.93 (0.89;0.99)** | **0.94 (0.89;0.99)** | 0.98 (0.93;1.03) | 1.00 (0.95;1.05) | **0.95 (0.90;1.00)** | 0.96 (0.91;1.02) |
| HH | 0.96 (0.88;1.05) | **0.86 (0.78;0.94)** | **0.84 (0.76;0.92)** | 0.94 (0.86;1.02) | 0.94 (0.86;1.02) | 0.97 (0.89;1.05) | 0.97 (0.90;1.06) |
| MV | 1.03 (0.93;1.14) | 1.00 (0.91;1.10) | 1.06 (0.96;1.16) | 1.05 (0.95;1.16) | **1.11 (1.01;1.21)** | **1.21 (1.11;1.32)** | 1.07 (0.97;1.17) |
| NI | 0.99 (0.95;1.04) | 0.97 (0.93;1.01) | 1.00 (0.96;1.04) | 0.98 (0.93;1.02) | 0.98 (0.94;1.02) | **0.94 (0.89;0.98)** | **0.95 (0.91;0.99)** |
| NW | **1.07 (1.04;1.10)** | **1.05 (1.02;1.08)** | **1.06 (1.03;1.09)** | 1.02 (0.99;1.05) | **1.04 (1.01;1.07)** | **1.05 (1.02;1.08)** | **1.03 (1.00;1.06)** |
| RP | **0.92 (0.86;0.98)** | 0.97 (0.91;1.03) | 0.95 (0.89;1.01) | 0.96 (0.90;1.02) | 0.95 (0.89;1.01) | 0.95 (0.89;1.01) | **0.92 (0.86;0.98)** |
| SH | 0.98 (0.90;1.05) | **0.92 (0.85;0.99)** | **0.89 (0.82;0.97)** | **0.92 (0.85;0.99)** | 1.02 (0.94;1.09) | 0.98 (0.91;1.06) | 0.94 (0.87;1.02) |
| SL | 0.94 (0.84;1.05) | 0.95 (0.85;1.06) | 0.96 (0.86;1.08) | **1.13 (1.02;1.25)** | 1.02 (0.91;1.13) | 1.10 (0.99;1.22) | 1.08 (0.97;1.20) |
| SN | **1.14 (1.08;1.20)** | **1.17 (1.11;1.23)** | **1.10 (1.05;1.16)** | **1.10 (1.04;1.16)** | **1.09 (1.03;1.16)** | **1.06 (1.01;1.12)** | 1.05 (0.99;1.11) |
| ST | 1.00 (0.93;1.08) | 1.02 (0.94;1.10) | 1.06 (0.99;1.14) | **1.09 (1.01;1.17)** | **1.13 (1.05;1.21)** | 1.04 (0.97;1.12) | 1.02 (0.95;1.10) |
| TH | 1.07 (0.98;1.15) | **1.08 (1.00;1.16)** | 1.05 (0.98;1.14) | 1.00 (0.92;1.07) | **1.08 (1.00;1.16)** | 0.99 (0.91;1.07) | 1.04 (0.96;1.12) |
| Urban | **1.03 (1.01;1.05)** | **1.02 (1.00;1.04)** | **1.03 (1.01;1.05)** | **1.02 (1.00;1.04)** | **1.03 (1.01;1.05)** | **1.04 (1.02;1.06)** | **1.03 (1.01;1.05)** |
| Rural | **0.98 (0.96;0.99)** | **0.98 (0.96;1.00)** | **0.97 (0.96;0.99)** | **0.98 (0.96;1.00)** | **0.97 (0.96;0.99)** | **0.97 (0.95;0.99)** | **0.97 (0.95;0.99)** |

*Statistically significant values in bold face.*

*Tab. A4-6 Descriptives and results for control variables of regression models on inpatient mortality (models for each calendar year separately and all years combined, respectively)*

|  | 2014 | 2015 | 2016 | 2017 | 2018 | 2019 | 2020 | 2014-2020 |
| --- | --- | --- | --- | --- | --- | --- | --- | --- |
| Included stroke cases | 248,453 | 253,498 | 257,432 | 256,584 | 252,863 | 252,843 | 238,384 | 1,760,057 |
| Number of clusters (hospitals) ^a^ | 1,252 | 1,235 | 1,209 | 1,189 | 1,158 | 1,149 | 1,118 | 8,310 |
| C-statistic | 0.781 | 0.779 | 0.781 | 0.782 | 0.782 | 0.783 | 0.784 | 0.781 |
| Covariates (OR) |  |  |  |  |  |  |  |  |
| Age (in years) | 1.06* | 1.06* | 1.06* | 1.065* | 1.06* | 1.07* | 1.07* | 1.06* |
| Female (ref.: male) | 1.17* | 1.18* | 1.16* | 1.173* | 1.20* | 1.17* | 1.16* | 1.17* |
| Type of stroke (ref.: ischaemic [I63]) ^b^ |  |  |  |  |  |  |  |  |
| Intracerebral haemorrhage (I61) | 5.72* | 5.78* | 6.05* | 6.15* | 6.32* | 6.36* | 6.81* | 6.15* |
| Subarachnoid haemorrhage (I60) | 4.95* | 5.16* | 5.58* | 5.29* | 5.46* | 5.59* | 5.40* | 5.35* |
| Stroke, not specified (I64) | 1.67* | 1.58* | 1.58* | 1.43* | 1.38* | 1.43* | 1.36* | 1.54* |
| Heart failure/ cardiomyopathy | 1.42* | 1.52* | 1.54* | 1.48* | 1.49* | 1.46* | 1.49* | 1.49* |
| Chronic ischaemic heart disease | 1.08* | 1.06* | 1.06* | 1.01 | 1.03 | 1.03 | 1.04 | 1.04* |
| Hypertension | 0.48* | 0.50* | 0.49* | 0.50* | 0.49* | 0.49* | 0.48* | 0.49* |
| Valvular heart diseases | 0.54* | 0.57* | 0.61* | 0.57* | 0.55* | 0.57* | 0.63* | 0.58* |
| Atherosclerosis of the extremities | 1.22* | 1.18* | 1.11* | 1.18* | 1.33* | 1.20* | 1.23* | 1.21* |
| COPD, asthma | 1.07 | 1.07* | 1.07 | 1.05 | 1.12* | 1.11* | 1.08* | 1.08* |
| Liver disease | 1.28* | 1.25* | 1.26* | 1.16 | 1.23* | 1.26* | 1.37* | 1.26* |
| Severe kidney disease or chronic renal failure | 1.09* | 1.07* | 1.03 | 1.04 | 1.00 | 1.01 | 0.95 | 1.03* |
| Diabetes mellitus | 0.98 | 1.01 | 1.02 | 1.04 | 1.06* | 1.01 | 1.02 | 1.02* |
| Obesity | 0.76* | 0.70* | 0.72* | 0.77* | 0.74* | 0.79* | 0.75* | 0.75* |
| Cachexia/ malnutrition | 1.37* | 1.20* | 1.23* | 1.24* | 1.25* | 1.35* | 1.25* | 1.27* |
| Coagulation disorder | 1.89* | 1.88* | 2.09* | 2.33* | 2.29* | 2.62* | 2.29* | 2.13* |
| Malignant neoplasm | 2.35* | 2.10* | 2.19* | 2.28* | 2.38* | 2.29* | 2.36* | 2.28* |
| Covid-19, confirmed | - | - | - | - | - | - | 2.94* | 2.90* |
| Year (ref.: 2014) |  |  |  |  |  |  |  |  |
| 2015 |  |  |  |  |  |  |  | 1.02 |
| 2016 |  |  |  |  |  |  |  | 1.01 |
| 2017 |  |  |  |  |  |  |  | 1.02 |
| 2018 |  |  |  |  |  |  |  | 1.03 |
| 2019 |  |  |  |  |  |  |  | 1.03 |
| 2020 |  |  |  |  |  |  |  | 1.10* |

* p <0.05; ^a^ in regression models for all years combined, hospital clusters are separated per calendar year, too, because hospital identifiers may change between hospitals over the years (e.g., through merging and acquisitions or via integration of previously separated hospital sites into one identifier); ^b^ codes for primary diagnoses as of the ICD-10-GM in brackets; ICD-10-GM codes of secondary diagnoses used for comorbidities (reference: no such secondary diagnosis code): heart failure/cardiomyopathy: 'I50' 'I110' 'I130' 'I132' 'I420' 'I426' 'I427' 'I428' 'I429', chronic ischaemic heart disease: 'I25', hypertension: 'I10' 'I119' 'I129' 'I139' 'I15', valvular heart diseases: 'I340' 'I342' 'I350' 'I351' 'I352' 'I050' 'I051' 'I052' 'I060' 'I061' 'I062' 'Q230' 'Q231' 'Q232' 'Q233', atherosclerosis of the extremities: 'I702', COPD, asthma: 'J41' 'J42' 'J44' 'J45' 'J47', liver disease: 'B18' 'I864' 'I982' 'K70' 'K73' 'K74' 'K760' 'K761' 'K765' 'K766' 'K767' 'Q446' 'Q447', severe kidney disease or chronic renal failure: 'I120' 'I131' 'I132' 'N03' 'N04' 'N05' 'N07' 'N08' 'N11' 'N12' 'N14' 'N15' 'N16' 'N18' 'N19' 'Z992', diabetes mellitus: 'E10' 'E11' 'E12' 'E13' 'E14', obesity: ‘E66’, cachexia/malnutrition: 'R64' 'R634' 'E43' 'E44', coagulation disorder: 'D66' 'D67' 'D680' 'D681' 'D682' 'D684' 'D685' 'D686' D688' 'D689' 'D691' 'D693' 'D694', malignant neoplasm: ‘C’, Covid-19, confirmed: 'U071';
OR – odds ratio, COPD – chronic obstructive pulmonary disease, Covid-19 – coronavirus disease 2019; ref. – reference category
